# Patients’ recommendations for a patient-centred public antiretroviral therapy programme in eThekwini, KwaZulu-Natal

**DOI:** 10.4102/sajhivmed.v18i1.677

**Published:** 2017-03-31

**Authors:** Delarise M. Mulqueeny, Myra Taylor

**Affiliations:** 1 School of Nursing and Public Health, University of KwaZulu-Natal, South Africa

## Abstract

**Background:**

The South African antiretroviral therapy (ART) programme, which is in its second decade of existence, includes many successes and challenges. This study provides patients’ recommendations to address the challenges they currently experience at four antiretroviral (ARV) clinics based in urban public hospitals in order to provide a patient-centred service.

**Objectives:**

To use patients’ recommendations to develop intervention strategies to improve patients’ experiences of the public ART programme.

**Method:**

A three-stage, sequential, mixed-method study was implemented. Stage 1 recruited five patients from the four sites to formulate and test a structured questionnaire prior to data collection. Stage 2 recruited a stratified random sample of 400 patients (100 from each hospital) to complete the administered structured questionnaire. Stage 3 purposively selected 12 patients (three from each of the four sites) to participate in in-depth audio-recorded interviews using an interview schedule.

**Results:**

The 412 patients prioritised six recommendations, which are as follows: waiting areas should be enclosed to protect patients from the elements (rain, sun, lightening, wind and cold); patients should not have to return their files to the main hospital or ARV clinic themselves; stable patients should collect their ARV drugs every three months; pharmacy opening and closing times should be revised to suit patients’ needs; HIV-positive patient representatives should be elected at each ARV clinic to address patients’ concerns and/or challenges to ensure that the programme could be more patient-centred and ARV clinic operating times should be extended to open later during weekdays and over weekends.

**Conclusion:**

Patients living with HIV have a valuable contribution to make in assessing service delivery and making recommendations to create a patient-centred healthcare environment, which will feasibly increase their adherence to ART.

## Introduction

In 2003, the South African Department of Health initiated a much welcomed antiretroviral therapy (ART) rollout programme for patients.^1^ Subsequent advancements in treatment as well as changes to the eligibility criteria have increased the availability of ART, resulting in people living with HIV (PLHIV) establishing families, living longer and enjoying meaningful employment.^2,3^ PLHIV require comprehensive HIV care, which includes management of opportunistic infections, nutritional deficiencies, antiretroviral (ARV) side effects and co-morbidities as well as assistance with psychosocial, spiritual and socio-economic challenges.^4,5^ This assistance must be ongoing for ARV adherence, to avoid drug resistance and promote retention, which is essential for the success of the ART programme and patients’ well-being.^6,7^

The public ART programme is perceived by many as successful.^8,9^ However, it is also affected by challenges,^10,11^ which include all PLHIV receiving quality treatment, adherence, sustaining patients on ART, closing the gap between urban and rural health services and addressing operational problems (strikes, staff and drug shortages, infrastructure challenges, overburdened staff and training and upscaling of staff).^12,13,14,15^ The national HIV Counselling and Testing campaigns^16^ and the South African Health Minister Aaron Motsoaledi’s announcement that as from September 2016 all HIV-positive patients could access ART irrespective of their CD4 count is likely to increase the number of patients attending the public ART programme and may further challenge the quality of patient care.^17,18,19^

## Relevance

This study reports patients’ recommendations based on their experiences, which serve as a patient-driven interventional component, to improve the local public ART programme. Involving patients in assessments of service quality and delivery is a growing trend and becoming a global phenomenon.^20^ Such a study is lacking in South Africa and could be beneficial to policy makers, programme directors and patients and serve as a catalyst to achieve patient-centred care within the current public ART programme and health systems, both locally and nationally.

## Objectives

This study investigated what could be implemented to enable the ART programme to be more patient-centred at four urban public hospitals. The objectives are to describe patients’ recommendations to improve the public ART programme, enhance treatment adherence and retention, reduce morbidity and mortality rates and ensure patient-centred care.

## Research design and method

A sequential, mixed-method, multi-site approach was employed to provide reliable and valid results.^21^ Initially, the study utilised structured questionnaires and thereafter face-to-face, in-depth interviews (IDIs) to provide recommendations to improve the quality of care at four ARV clinics, based in hospitals within eThekwini District, KwaZulu-Natal. The reason for the study taking place in this province is that it has the second largest population in South Africa and is at the epicentre of the South African HIV epidemic.^22,23,24^

Initially, five experienced and articulate ART patients of both sexes and different ages, a minimum of one from each facility, who did not participate in further data collection, described their experiences and the challenges they encountered and together formulated a total of 41 recommendations to ensure a patient-centred ART programme at their facility. These were included in the questionnaire.

A stratified random sample of patients (400), 100 from each clinic from four public hospitals in the eThekwini District, participated in the administered structured questionnaires. Every fourth patient visiting the clinic was systematically and randomly selected starting with the first patient attending the clinic daily. The quantitative sample comprised 161 men and 239 women of different ages, ethnicity and sexual orientation. Twelve respondents (three from each site) for the IDIs were purposively recruited to achieve gender, race and sexual orientation representation. Six female and six male patients, aged between 18 and 67 years, participated in the interviews, with seven respondents identifying as heterosexual, four as gay and one as bisexual.

All 412 HIV sero-infected patients met the study criteria as they were aged 18 years or older and had accessed the ART programme at one of the four ARV clinics for at least one year or more. Completion of all the questionnaires and interviews took place in and around the outpatient ARV clinics within the hospital grounds.

Informed consent and confidentiality was explained to ensure voluntary participation in the study. No refusals were documented, and all 412 respondents signed informed consent forms prior to the data collection process. This took place from August to November 2015.

All the questionnaires were administered by the principal investigator (PI) and two research assistants who were fully conversant in Zulu, Afrikaans and English. The patients requested the interviews be held in English; however, the trilingual research assistants were also present during the process. No language challenges were reported. The researcher conducted the IDIs with an interview schedule and utilised probes where and when necessary.

### Data analysis

Questionnaires were checked prior to the patients leaving the site, and the data were entered into Epidata software and analysed utilising the Statistical Package for the Social Sciences (SPSS 22) software. The interview transcripts were read several times to ensure a thorough understanding of the information and context. Open-coding techniques were used for the content analysis of each transcript to identify themes and sub-themes based on recurring ideas and concepts.^25^

### Reliability and validity

A sequential, mixed-method design was appropriate to provide reliable, valid results, which Johnson and Onwuegbuzie^26^ describe as important as ‘research approaches should be mixed in ways that offer the best opportunities for answering important research questions’ (p. 16). To ensure all data were accurately captured, dual recording took place during the interviews. The transcripts were read several times by the researcher and coded using thematic analysis to identify differences and similarities of respondents’ answers to ensure the dependability and trustworthiness of the data. Coded questionnaires and interview transcripts ensured anonymity, which provided valid results. The process was continuously discussed and checked by the co-author.

### Potential benefits and hazards

All respondents were allowed to ask questions regarding the questionnaires and interviews prior, during and subsequent to data collection taking place, to ensure all respondents understood the research process. The sensitive nature of the research study dictated that confidentiality and privacy were respected at all times. Patients were offered follow-up sessions with a social worker or counsellor to avoid any psychological or emotional harm.^27^ To respect busy ARV clinics, continuous communication with the clinic staff took place.^28^ All completed anonymous questionnaires, electronic records, tape recordings and transcripts were stored in a locked cupboard for the duration of the study and will be destroyed five years after study completion.

#### Ethical consideration

The Biomedical Research Ethics Committee (BFC089/15) and the KwaZulu-Natal Department of Health (HRKM158/15) granted ethical clearance, while site clearance was obtained from the four hospital managers prior to the commencement of the study.

## Results

### Change, the only constant to ensure patient-centred care

**FIGURE 1:**
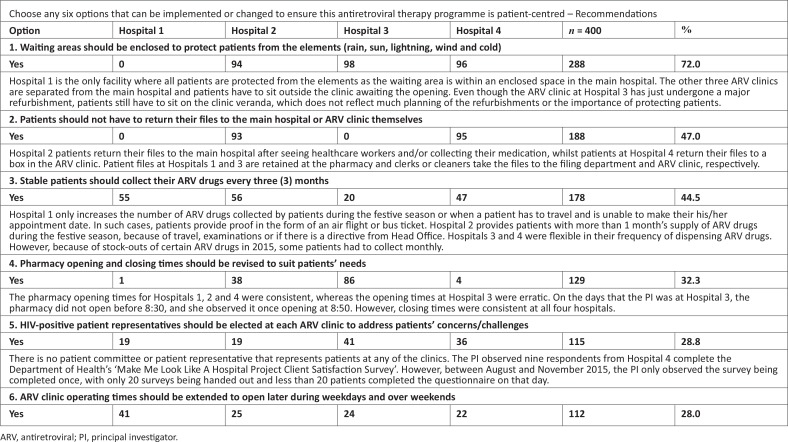
Patient recommendations.

Five patients from public ARV clinics, together with the PI, formulated 41 recommendations, to ensure that the ART programme at all four hospitals is patient-centred. Patients were required to choose six primary recommendations for the purpose of this study. The quantitative data are presented in Figure 1.

### Recommendations from the in-depth interviews

The six most popular recommendations from the IDIs were similar to those chosen by respondents who answered the questionnaires.

#### Physical space and infrastructure

Respondents reported discomfort with open waiting areas as follows:

‘They should put an urn outside so we can make ourselves some tea in winter or provide us with sandwiches while we wait outside.’ [Participant 12, female, 57 years]

‘We have been sitting like this for too long. This waiting area needs to be closed up.’ [Participant 7, male, 38 years]

‘Government and staff should work with private companies to find money to improve this clinic.’ [Participant 9, male, 50 years]

#### Patient files

The recommendations below expose an inadequate filing system:

‘The filing system must be improved because our files keep getting lost.’ [Participant 5, female, 67 years]

‘They should invest in a computerised filing system.’ [Participant 11, male, 40 years]

‘Hiring qualified staff in the filing department would help.’ [Participant 1, female, 62 years]

#### Antiretroviral drugs collection

Outsourcing of pharmacies, flexibility of pharmacy operation times and dispensing processes and reduction in the costs of ARV drugs collection are highlighted below:

‘It would be so much better if our meds were delivered to our home or work.’ [Participant 2, female, 26 years]

‘The quicker they outsource the ARV pharmacy the better. It would be much better to collect our medication at private pharmacies such as Dischem or Clicks as we can collect on weekends and after work.’ [Participant 5, female, 67 years]

‘Surely it would save the staff time, money and irritation if they give us our medication once every three months. I know all the patients would be happy because we would save taxi fare.’ [Participant 6, male, 18 years]

#### Patient representation and satisfaction

Respondents’ need for patient involvement, engagement and representation are expressed below:

‘I feel we should have a representative, representing us. We should do anonymous forms and hand it in outside this clinic.’ [Participant 1, female, 62 years]

‘If we had one of our own representing us I think the service would improve for us.’ [Participant 2, female, 26 years]

‘Patients should be involved because patients tell other patients the truth, not staff. They need our input so they should employ us as patient therapists because we are the experts.’ [Participant 3, male, 46 years]

‘A representative should sit in on hospital meetings so our problems are put forward.’ [Participant 6, male, 18 years]

‘We need to be involved in this programme so that service improves all the time.’ [Participant 8, female, 29 years]

‘Patients must be represented so we know and understand our rights.’ [Participant 9, male, 50 years]

‘I don’t think a patient rep will make a difference, we are just HIV patients.’ [Participant 10, male, 46 years]

‘Even though we receive good service from the clinic staff I think it is important for a representative to discuss the pharmacy story. They should hire patients as role-models.’ [Participant 7, male, 38 years]

These responses emphasise patients’ need for a trustworthy, engaging and empowering environment and a programme that promotes patients’ self-worth and rights. All these recommendations promote and embody patient-centred care.

#### Pharmacy operational times

The responses below articulate patients recommending outsourcing of the ARV pharmacies and revision of operational times:

‘They should close this pharmacy and let us go to private chemists to collect.’ [Participant 4, male, 35 years]

‘If they don’t want to change the weekday times they should let us collect on the weekend or from private chemists.’ [Participant 8, female, 29 years]

‘The pharmacy is an area of concern at this clinic. If they can sort it out, then this would be a clinic that other medical staff could learn a thing or two from the staff at this clinic.’ [Participant 7, male, 38 years]

The latter response highlights the respondents’ satisfaction with service delivery and even suggests learning opportunities for other ARV clinic staff. This latter respondent accesses treatment at Hospital 3, which has inconsistent pharmacy opening times.

#### Antiretroviral clinic operational times

Extension of clinic operational times and inclusion of signage to suit patients’ needs are recommendations provided below:

‘We should be able to attend a clinic after hours. [Participant 4, female, 35 years]

‘Signs should be put up of how long it’s going to take us in this clinic.’ [Participant 5, female, 67 years]

‘Weekends are the solution as most working people are off work.’ [Participant 10, male, 46 years]

‘All clinics should open at 7 o’clock and close at 5 pm so that we can be seen before or after work.’ [Participant 11, male, 40 years]

Worth noting was that the majority of the patients arrived at the hospital between 4:00 and 7:00, which entailed them leaving their homes very early to meet their scheduled 7:00 appointments. Respondents required ARV clinics operational times to be extended to open earlier, close later and include weekends.

## Discussion

Patient-centred care places patients at the centre of their healthcare journey.^29^ Hence, patients recommending improvements and changes to ensure a patient-centred ARV clinic is both empowering and inclusive to the patient healthcare dynamic.^30^ The six most popular recommendations focus on improvement to the physical space and infrastructure, patients’ files, ARV drug collection frequency, patient representation and satisfaction, pharmacy operational times and the ARV clinic operational times.

### Physical space and infrastructure

The majority of the questionnaire respondents (72%) recommended enclosed waiting areas, which concurs with an evaluation of the ART programme in KwaZulu-Natal, South Africa, which highlighted the ‘mismatch between supply and demand’.^31^ A comfortable safe environment where patients are not concerned about the elements and being seen by others should be a priority for all sites as is the case with Hospital 1.^32^

### Patient files

The results concur with the findings of an assessment of public ARV clinics conducted between June and November 2009 and another study conducted at a Johannesburg clinic, which highlighted challenging filing systems as new files were opened and such challenges lead to loss to follow-up.^33,34^ An electronic or improved filing system could improve patient turnaround time and lessen the opportunity for files being lost. The study highlighted the inconsistency and variability of services and processes between sites, which requires further interrogation.

### Antiretroviral drug collection and pharmacy operational times

ARV stock-outs, transportation costs and lengthy waiting times are challenges that patients are confronted with when collecting their ARV drug and other chronic medication. The study results concur with a study conducted at a public hospital, which concluded that ARV drugs are provided free of charge; however, the cost of attaining them (transport, time off work) is high and could affect adherence.^35,36^ The introduction of the Central Chronic Medicine Dispensing and Distribution Programme (CCMDD) is an attempt at alleviating such challenges. This programme allows patients to collect their chronic medication from designated pharmacies and healthcare facilities. These facilities operate after hours and on weekends and would be meaningful and cost-effective for patients on ARV drugs, as they would be able to access their medication nearer to their homes and/or workplace at times that are convenient for them.^37^ However, the data collection process took place in 2015 and this intervention had not been implemented at the four study sites. During 2016 some sites have introduced the programme. Hence a follow-up study on the impact of the CCMDD programme might yield different results.

### Patient representation and satisfaction

Respondents viewed themselves as ‘patient therapists’, ‘role-models’ and ‘experts’ who should be involved and/or employed at ARV clinics to assist other patients and play an active role in improving systems and processes to enhance patient care.^38^ These findings concur with a study conducted at 18 Sub-Saharan health facilities to assess the degree of patient involvement at HIV care hospitals, which concluded that patient involvement was a necessity towards improving service delivery.^34^ A Malawian study also highlighted the benefits of involving PLHIV in treatment programmes.^39^ Our study results also highlighted patients’ internalised and healthcare workers’ stigma and disrespect, which contradicts the findings of Kieft et al. 2014.^40^

### Antiretroviral clinic operational times

Most patients were in the ARV clinic for between one and two hours from the time they entered to the time they exited. This timeframe did not include collecting their ARV drugs as three ARV pharmacies are separate from the ARV clinics. The refurbishments at Hospital 2’s ARV clinic included a pharmacy inside the clinic, which was unique and saved patients’ time. The pharmacy and clinic operational times have been documented as challenges to patients in other studies.^41,42^ Hence, patients’ recommendations of flexible and extended operational hours, which include earlier and later closing times and weekends, could be beneficial. The rollout of the Central Chronic Medicine Dispensing (CCMD) and implementation of flexible, extended hours could improve patients’ experiences.

Some recommendations can be implemented at the hospital level, whereas others require provincial and national intervention in terms of budgetary and human capital resources. These would promote patient-centred care through strengthened patient–provider relationships; improved and extended pharmacy and operational times; warm, nurturing clinics; continuous improvement; qualified, patient-friendly and respectful staff; empowering and engaging systems; reciprocal communication; improved outsourcing services; patient representation and satisfaction; destigmatised clinical environments; and updated filing systems and processes. The recommendations further provide opportunities for staff to learn best practices from each other and other clinics to improve their systems and processes.

However, inasmuch as these six recommendations did not prioritise waiting times, waiting areas, staff attitudes, patient–provider relationships, staff training, stock-outs and financial assistance, the other recommendations did and are discussed in subsequent papers. The patients’ six recommendations and the 35 that are included in Appendix 1 would result in a patient-centred clinic that patients approve of and signify an ideal ARV clinic.

#### Limitations of the study

The study was conducted at four hospitals within eThekwini district; therefore, more studies at other hospitals and Primary Health Care (PCH) facilities could yield further recommendations for improvement of the current ART programme in KwaZulu-Natal.

#### Recommendations

Staff perceptions and experiences of the public ART programme as well as their recommendations for patient-centred ARV clinics could be explored in future studies.

## Conclusion

As patients are the cornerstone of the South African ART programme, it is important and relevant to understand their needs 12 years after the rollout of the public ART programme. The findings are concerning for sites within a large metro; however, room for change and improvements to the programme were highlighted. The study also emphasised that the patients were prepared to assist in improving the status quo by offering recommendations that could be feasibly implemented to improve the current ART programme. The results presented patients’ willingness to participate as ‘patient therapists’, ‘role-models’ and ‘expert’ patients. This study concurs with other relevant studies regarding patient experiences of the public ART programme and healthcare system but goes further to provide recommendations for improvement.

## Appendix 1

**TABLE 1-A1:** Other recommendations.

Recommendation	Option	Hospital 1	Hospital 2	Hospital 3	Hospital 4	*n* = 400	%
**Space, infrastructure, service and processes**							
Food and refreshments should be served while patients are waiting to be attended to	Yes	11	15	36	28	90	22.5
Pill counts should be stopped when patients are deemed stable by the doctor	Yes	0	1	0	53	54	13.5
Registration and admin processes should be streamlined and made patient-friendly	Yes	39	7	0	1	47	11.8
Opening times for cashiers and ARV clinics should be regulated to ensure that they open at the same time every day	Yes	30	0	1	5	36	9.0
Patients should have their blood pressure, sugar levels and weight checked at all ARV clinics	Yes	32	0	0	0	32	8.0
ARV patients should only register at the ARV clinic	Yes	11	18	0	0	29	7.3
Turnaround time for routine and emergency blood results should be improved so patients do not wait so long	Yes	5	3	18	0	26	6.5
Patients should be allowed to make and change their appointments over the telephone	Yes	17	8	0	0	25	6.3
It should be mandatory that health talks are held daily and are conducted in isiZulu and English at this ARV clinic	Yes	2	7	2	0	11	2.8
IsiZulu and English HIV support groups should be formed at all ARV clinics	Yes	2	2	7	0	11	2.8
**Filing systems and processes**							
Barcoded patient cards and files should be implemented so patients swipe instead of waiting in queues	Yes	26	22	29	11	88	22.0
Filing systems should be improved to avoid files being lost	Yes	27	10	0	1	38	9.5
Separate ARV patient files should be kept at the ARV clinic to avoid being lost in the main hospital filing department	Yes	20	0	0	1	21	5.3
**Staff, supervision and training**							
Supervisors should conduct ad hoc checks to ensure staff are doing their job and treating patients professionally	Yes	60	24	20	5	109	27.3
Dieticians should be based in all ARV clinics to advise patients about their dietary requirements	Yes	22	12	40	13	87	21.8
Staff meetings should take place in the afternoons	Yes	20	34	17	11	82	20.5
A minimum of two fully qualified doctors should be permanently on duty at this ARV clinic to ensure swift service	Yes	0	0	1	75	76	19.0
Staff should not be allowed to use their personal cell phones and tablets during working hours	Yes	16	12	11	3	42	10.5
Social workers should be stationed at each ARV clinic to address patients’ concerns and challenges	Yes	14	9	2	8	33	8.3
Vacant positions should be filled as soon as possible to ensure clinics are adequately staffed	Yes	0	7	6	14	27	6.8
Regular training should take place so that staff are up-to-date about HIV treatment, policies and guidelines	Yes	8	3	7	2	20	5.0
Staff should receive regular training and updates about the LGBTI community and diversity	Yes	8	5	2	3	18	4.5
Staff supervisors should ensure that staff commence work on time and adhere to the stipulated tea and lunch times	Yes	7	2	0	2	11	2.8
Automated clock card systems should be placed at each ARV clinic to monitor staff attendance	Yes	0	2	1	1	4	1.0
Staff lunch and tea times should be staggered to ensure there are always sufficient staff on duty	Yes	2	1	1	0	4	1.0
**Communication and signage**							
IsiZulu and English legible signs and flow charts should be put up	Yes	1	0	5	1	7	1.8
IsiZulu and English legible signs should be put up to inform patients when staff are on tea and lunch	Yes	2	2	2	0	6	1.5
**Pharmacy**							
ARV scripts should be saved in the pharmacy’s computer system to avoid patients having to fetch and carry files	Yes	27	14	41	14	96	24.0
Pharmacy operational and waiting time signs should be put up outside the pharmacy so that patients are aware of them	Yes	0	0	12	1	13	3.3
**Patient representation and satisfaction**							
Patients should form part of ARV clinic committees to ensure they are represented on matters that affect patients	Yes	31	28	19	17	95	23.8
Patients should complete anonymous questionnaires after each visit regarding the service they received	Yes	11	11	10	6	38	9.5
**Outsourcing and networking**							
Antiretroviral treatment programmes should be outsourced to private doctors/hospitals to reduce the strain on the public health system	Yes	18	3	16	10	47	11.8
Private pharmacies such as Dischem, Clicks and Sparksport should be allowed to dispense ARV drugs	Yes	11	6	17	13	47	11.8
Private laboratories should be used for routine blood tests and blood results should be linked to the ARV clinic	Yes	3	1	3	0	7	1.8
ARV drugs should be delivered to patient’s homes	Yes	1	2	3	0	6	1.5

ARV, antiretroviral; LGBTI, Lesbian, gay, bisexual, transgender, and intersex.

Note: For inclusivity, the other 35 recommendations have been divided into themes.
